# Renal Function in Hepatosplenic Schistosomiasis – An Assessment of Renal Tubular Disorders

**DOI:** 10.1371/journal.pone.0115197

**Published:** 2014-12-22

**Authors:** Daniella Bezerra Duarte, Lucas Alexandre Vanderlei, Raianne Kívia de Azevêdo Bispo, Maria Eliete Pinheiro, Geraldo Bezerra da Silva Junior, Alice Maria Costa Martins, Gdayllon Cavalcante Meneses, Elizabeth De Francesco Daher

**Affiliations:** 1 Department of Internal Medicine, School of Medicine, Federal University of Alagoas, Maceió, AL, Brazil; 2 Department of Internal Medicine, Post-Graduation Program in Medical Sciences, School of Medicine, Federal University of Ceará, Fortaleza, CE, Brazil; 3 School of Medicine, Post-Graduation Program in Collective Health, Health Sciences Center, University of Fortaleza, Fortaleza, CE, Brazil; 4 Department of Pharmacy and Clinical Analysis, Federal University of Ceará, Fortaleza, CE, Brazil; University of Bari Aldo Moro, Italy

## Abstract

**Background:**

Renal involvement in *Schistosoma mansoni* infection is not well studied. The aim of this study is to investigate the occurrence of renal abnormalities in patients with hepatosplenic schistosomiasis (HSS), especially renal tubular disorders.

**Methods:**

This is a cross-sectional study with 20 consecutive patients with HSS followed in a medical center in Maceió, Alagoas, Brazil. Urinary acidification and concentration tests were performed using calcium chloride (CaCl_2_) after a 12-h period of water and food deprivation. The biomarker monocyte chemoattractant protein 1 (MCP-1) was quantified in urine. Fractional excretion of sodium (FE_Na+_), transtubular potassium gradient (TTKG) and solute-free water reabsorption (TcH_2_O) were calculated. The HSS group was compared to a group of 17 healthy volunteers.

**Results:**

Patients' mean age and gender were similar to controls. Urinary acidification deficit was found in 45% of HSS patients. Urinary osmolality was significantly lower in HSS patients (588±112 vs. 764±165 mOsm/kg, p = 0,001) after a 12-h period of water deprivation. TcH_2_O was lower in HSS patients (0.72±0.5 vs. 1.1±0.3, p = 0.04). Urinary concentration deficit was found in 85% of HSS patients. The values of MCP-1 were higher in HSS group than in control group (122±134 vs. 40±28 pg/mg-Cr, p = 0.01) and positively correlated with the values of microalbuminuria and proteinuria.

**Conclusions:**

HSS is associated with important kidney dysfunction. The main abnormalities found were urinary concentrating ability and incomplete distal acidification defect, demonstrating the occurrence of tubular dysfunction. There was also an increase in urinary MCP-1, which appears to be a more sensitive marker of renal damage than urinary albumin excretion rate.

## Introduction

*Schistosoma mansoni* infection remains an important public health problem. This chronic parasitic disease affects about 200 million people worldwide [1]. Renal involvement in schistosomiasis is described mainly by glomerular involvement [2], [3], [4], [5]. Schistosomal glomerulopathy is associated with the hepatosplenic form of the disease; however, it has also been observed in the hepatointestinal form [6], [7]. Its incidence varies between 5–6%, increasing to 15% when considering only patients with the hepatosplenic form [8]. The immunological nature of glomerular involvement in schistosomiasis is well established [5], [9], [10]. The most frequently reported histological types are chronic membranoproliferative glomerulonephritis and focal segmental glomerulosclerosis, usually with nephrotic syndrome [11], [12], [13], [14]. There are other important factors, in addition to parasite antigens, which seem to contribute to the pathogenesis of glomerular disease in schistosomiasis, such as the collateral circulation of the portal system due to the degree of hepatic involvement, the inefficiency of the hepatic macrophage system, the severity and duration of infestation, racial and genetic factors [13], [15].

The search for new renal biomarkers is extremely important, as they may provide early diagnosis of renal disorders, allowing the adoption of measures to prevent progression to end-stage renal disease. The Monocyte Chemotactic Protein-1 (MCP-1) is one of the recently studied new biomarkers. This protein is expressed in injury and inflammation sites and directs the recruitment of macrophages, which bind to chemokine receptors to promote macrophage adhesion and chemotaxis [16]. MCP-1 has been correlated with urinary levels of albuminuria in diabetic nephropathy patients and also in other primary and secondary glomerulopathies [17], [18]. Recent Brazilian study found high levels of the urinary biomarker MCP-1 in patients with asymptomatic schistosomiasis [19].

To date, there is no study about renal tubular disorders in patients with hepatosplenic schistosomiasis. Thus, the aim of this study was to investigate renal tubular disorders in patients with hepatosplenic schistosomiasis (HSS) and to investigate its association with MCP-1.

## Materials and Methods

### Patients

This is a cross-sectional study of 20 consecutive patients with clinical and laboratory diagnosis of HSS, undergoing infectious disease consultation in public health centers in the Northeast of Brazil, from August 2012 to August 2013. Exclusion criteria were age less than 18 or greater than 65 years, hypertension (systolic blood pressure ≥140 mmHg and/or diastolic blood pressure ≥90 mmHg), diabetes mellitus (fasting glucose levels greater than 100 mg/dL and/or treatment for diabetes), heart failure, diuretic use within the last 15 days, systemic lupus erythematosus or another collagenosis, recurrent urinary tract infection or history of previous renal disease (glomerular filtration rate ≤60 mL/min, nephrolithiasis and use of nephrotoxic drugs). Liver disease of different etiologies (viral or alcoholism) and decompensated schistosomiasis within the last 6 months, with ascites or gastrointestinal bleeding were also excluded. Serological tests for hepatitis B and C were performed in all patients. All patients had been treated at least once in their lifetimes with a specific antiparasitic drug and the time interval between treatment and study enrollment was at least one year. The drugs used were praziquantel (40–50 mg/kg single dose) or oxaminiquine (15 mg/kg single dose). The patients were compared to a control group that consisted of 17 healthy volunteers, all blood donors. They all were tested for schistosomiasis.

### Ethics Statement

The study protocol was approved by the Ethical Committee of the Institution (Federal University of Alagoas, protocol 004228-2011-81). Patients were included in the study only after signing the informed consent form.

### Hepatosplenic schistosomiasis diagnosis

Schistosomiasis diagnosis was suspected based on epidemiological and clinical data and confirmed by detection of *Schistosoma mansoni* eggs in feces, rectal or liver biopsy. Clinical diagnosis of HSS was based on the presence of hepatomegaly and splenomegaly in physical or ultrasonographic examination, periportal fibrosis, increased portal vein diameter, previous history of gastrointestinal bleeding, presence of esophageal varices at endoscopy or history of splenectomy. The mean time of diagnosis was determined from medical records.

### Clinical and laboratorial parameters

At the medical consultation, signs and symptoms were evaluated and the following aspects were recorded: age, gender, race, previous chronic diseases (heart failure, arterial hypertension or diabetes mellitus), time of disease, use of other concomitant drugs, systolic and diastolic blood pressure, heart rate, weight, height and body mass index (BMI). The following laboratory parameters were evaluated: complete blood count, fasting blood glucose, plasma creatinine (P_cr_), urea (P_Urea_), arterial pH, bicarbonate (Bic), sodium (P_Na+_), potassium (P_K+_), calcium (P_Ca2+_), phosphorus (P_P_), magnesium (P_Mg2+_) and chloride (P_Cl_-), serum albumin and globulin, aspartate aminotransferase (AST), alanine aminotransferase (ALT), gamma-glutamyl transferase (GGT), total bilirubin and fractions, alkaline phosphatase (ALP), international normalized ratio (INR), complement (laboratory C3 and C4), 24-h urinary creatinine, sodium, potassium, magnesium, chloride, proteinuria (U_prot_V), urinalysis, urinary MCP-1 and microalbuminuria. The Child-Pugh score was used to assess liver function of patients [20], [21].

### Renal function evaluation

Glomerular Filtration Rate (GFR) was measured in 24-hour urine collection and was considered abnormal when ≤60 ml/min/1.73 m^2^. All patients were submitted to a 12-h water and food deprivation. Fractional excretion of sodium (FE_Na+_), magnesium (FE_Mg++_) and potassium (FE_K+_), transtubular potassium gradient (TTKG) and solute-free water reabsorption (TcH_2_O) were calculated by standard formulae. Microalbuminuria was determined by 24-h urine collection and abnormal values were >30 mg/day. MCP-1 was determined in the first urine sample (T0) and the values corrected by urinary creatinine.

Urinary concentrating ability was evaluated by the ratio between urinary and plasma osmolality (U/P_osm_) after a 12-h water and food deprivation. A baseline sample (T_0_) was collected before the administration of an intranasal spray of DDAVP [22] (desmopressin acetate, 20 mcg) and a second sample was collected 4 h (T_4_) later.

Urinary acidification was evaluated by measuring urinary pH (U_pH_) at baseline (T_0_) and 4 h (T_4_) after ingestion of CaCl_2_, 2 mEq//kg of body weight [23]. Metabolic acidosis induced by calcium chloride (CaCl_2_) load was documented by a decrease in serum HCO_3_– concentrations >3 mmol/1 and a pH <7.35. Failure to decrease urinary pH to <5.5 after CaCl_2_ load was considered consistent with some form of distal renal tubular acidosis (RTA). All tubular function tests were also performed in the control group.

### Analytical methods

Plasma creatinine was measured by Bonsness and Taussky method. Urea was determined by the colorimetric uricase method. Sodium and potassium were measured by flame photometry. Magnesium, calcium and phosphorus were determined by the colorimetric method. Chloride was determined by the ion selective electrode method. Urinalysis was determined by qualitative proteinuria, glycosuria and ketonuria, using reagent strips (Labistix, Lab. Ames, Miles do Brasil). Urinary sediment was analyzed by phase microscopy. Proteinuria was measured in 24-h urine samples by the turbidimetric method after precipitation with 1% sulfosalicylic acid (upper normal value 0.15 g/day). Microalbuminuria was measured by immunoturbidimetric methods (Tinaquant Roche). Osmolality was assessed by freezing-point depression. Blood pH and bicarbonate were determined in a pH blood gas system (AVL compact-1, Medical Instruments). Urinary pH was measured with a pH-meter (pG1800, GEHAKA). C3 and C4 complement fractions were measured by immunoturbidimetry. Total serum albumin and globulin were measured by protein electrophoresis. Urinary MCP-1 measurement was performed through ELISA using the R&D Systems kit, Inc (Minneapolis, MN, USA) and the values corrected by urinary creatinine.

### Statistical methods

HSS patients were compared with the control group. The chi-square, Pearson, likelihood ratio and Fisher's exact test were applied to measures of association and homogeneity in the distribution of categorical data. To verify the normality of distribution of continuous variables, the Kolmogorov-Smirnov test was used. The Levene test was used to compare variability. In the condition of data normality, the comparison between two means was made by Student's *t* test. In the case of non-normal data, the Mann-Whitney test was applied. Pearson's correlation test was used to compare two numeric variables. Pearson' or Spearman' correlation coefficients, when appropriate, were used to test the association between continuous variables. Data were expressed as mean ± SD. P<0.05 was considered statistically significant. SPSS software for Windows, release 16.0 (SPSS Inc. Chicago, USA) was used in all the analyses.

## Results

Of the 53 patients followed in the outpatient clinics with a diagnosis of hepatosplenic schistosomiasis from August 2012 to August 2013, 11 were excluded due to age older than 65 years, 5 due to diabetes mellitus type 2, 4 due to previous systemic arterial hypertension, 3 due to previous nephrolithiasis, 4 due to chronic viral hepatitis, 3 due to previous chronic kidney disease and 3 due to alcoholic liver cirrhosis. Twenty patients who agreed to participate in the study were included. The mean age of the patients was 42.2±9.2 years (range 32–57), 50% of each gender. All patients had epidemiological criteria for schistosomiasis. The mean educational level was 3.9±3.0 years of study. Mean time of diagnosis was 15.5±11.5 years. History of *Schistosoma mansoni* eggs in feces was identified in 75% and diagnosis by liver biopsy was carried out in 25% of patients. All patients had splenomegaly and 70% had undergone splenectomy within a time period of 10.5±10 years. Hepatomegaly, however, was present in only 20% of cases. All patients had evidence of periportal fibrosis at ultrasonography. Intestinal symptoms were found in 30% of patients. Esophageal varices were present in 95% of patients and 55% of them had experienced previous episodes of gastrointestinal bleeding. Other demographic and clinical characteristics are shown in Table 1.

**Table 1:** Demographic and clinical characteristics of the study population.

	Hepatosplenic schistosomiasis (*n* = 20)	Controls (*n* = 17)	*P*
Age, years (Mean ± SD)	42.2±9.2	34.8±15.2	0.09
Male, %	50	53	1.0
Systolic blood pressure, mmHg	114±18	122±9	0.21
Diastolic blood pressure, mmHg	73±10	79±5	0.07
Time of disease, years	15.5±11.5	---	---
Splenomegaly, %	100	---	---
Prior splenectomy, %	70	---	---
Periportal fibrosis, %	95	---	---
Esophageal varices, %	95	---	---
Previous upper gastrointestinal bleeding, %	55	---	---
Intestinal disorders, %	30	---	---
Hepatomegaly, %	20	---	---
Positive stool examination for *S. mansoni* eggs, %	75	---	---
Positive liver biopsy, %	25	---	---

Data are shown as mean ± SD or percentages. Significant P <0.05 *vs.* control by Student' *t* and Fisher's exact tests.

All patients had Child-Pugh score A. Anemia was observed in one patient (5%), leukopenia in 4 (20%) and thrombocytopenia in 6 (30%). Platelet levels were lower in the HSS group compared with the control group (195,520±107,290 *vs.* 270,660±34,092/mm^3^, *P* = 0.01). Hypergammaglobulinemia was present in 65% of patients and hypocomplementemia in 10 (50%). There was no correlation between complement levels and renal function parameters. The results of general laboratory parameters are summarized in Table 2.

**Table 2:** General laboratory parameters of hepatosplenic schistosomiasis patients and controls.

	Hepatosplenic schistosomiasis (*n* = 20)	Controls (*n* = 17)	*P*
Hemoglobin, *g/dL*	13.9±1.4	14.1±1.3	0.80
Hematocrit, *%*	42.4±4.2	42.4±4.5	1.0
Leukocytes,/*mm^3^*	6,098±2,760	6,446±479	0.60
Platelets,/*mm^3^*	195,520±107,290	270,660±34,092	0.01
P_Na,_ *mEq/L*	139±3.0	138±3.9	0.15
P_K_, *mEq/L*	4.4±0.4	4.3± 0.4	0.41
P_Ca_, *mg/dL*	9.7±0.6	9.2±0.3	0.05
P_P_, *mg/dL*	3.2±0.5	3.6±0.6	0.15
P_Cl_, *mEq/L*	103±3.2	107±7.6	0.43
P_Mg_, *mg/dL*	1.9±0.2	1.9±0.2	0.52
C3, *mg/dL*	88±28	---	---
C4, *mg/dL*	19±9	---	---
AST, *UI/L*	41±13	---	---
ALT, *UI/L*	37±12	---	---
Alkaline phosphatase, *UI/L*	120±86	---	---
GGT, *UI/L*	104±82	---	---
Total bilirubin, *mg/dL*	0.94±0.46	---	---
Direct bilirubin, *mg/dL*	0.23±0.25	---	---
Indirect bilirubin, *mg/dL*	0.71±0.25	---	---
Total protein, *g/dL*	8.1±0.4	---	---
Albumin, *g/dL*	4.3±0.4	---	---
Globulin, *g/dL*	3.8±0.55	---	---
INR	1.28±0.18	---	---

Values are expressed as mean ± SD. Significant P<0.05 *vs* control by Student *t* test. P_Na+_, plasma sodium; P_K+_, plasma potassium; P_Ca2+_, plasma calcium; P_P_, plasma phosphorus; P_Cl_-, plasma chloride; PMg2+, plasma magnesium AST, aspartate aminotransferase; ALT, alanine aminotransferase; GGT, gamma glutamyl transferase; INR, international normalized ratio.

### Hepatosplenic schistosomiasis patients have higher GFR and altered tubular function in comparison with the controls

The comparison between the HSS patients and the control group showed no differences in age, gender, body mass index, systolic and diastolic blood pressure (Table 1). As seen in Tables 3 and 4, HSS patients had higher GFR (130±38 *vs.* 103±16 mL/min/1.73 m^2^, *P* = 0.01). Glomerular hyperfiltration (GFR>120 mL/min/1.73 m^2^) was observed in 8 (40%) HSS patients. GFR≤60 ml/min/1.73 m^2^ was not observed in any patient. Urinary concentrating ability defect, as demonstrated by a lower U_Osm_ after desmopressin (DDAVP) administration (588±112 *vs* 764±165 mOsm/Kg, *P* = 0.001) and a lower U/P_Osm_ ratio (2.05±0.40 *vs.* 2.66±0.55, *P*<0.001) was observed when comparing HSS patients and controls. No increase of U_Osm_ after DDAVP was observed in 17 (85%) patients. Urinary pH did not decrease to 5.5 or less in response to acid-loading with CaCl_2_ in 9/20 (45%) of patients. Levels of serum bicarbonate (HCO_3_-) were lower in HSS patients. Arterial pH was similar in the two groups before and after the acid-loading with CaCl_2_. There was no difference between the HSS group and the control group regarding FE_Na+_, FE_Mg++_, FE_K+_ and TTKG. The levels of TcH_2_O were lower in HSS patients than in the control group (0.72±0.5 *vs.* 1.1±0.3, *P* = 0.04).

**Table 3:** Comparison of urinary concentration and acidification tests in hepatosplenic schistosomiasis patients and controls.

	Hepatosplenic schistosomiasis (*n* = 20)	Controls (*n* = 17)	*P*
*U*_osm_ T4 (mOsm/kg.H_2_O)	588±112	764±165	0.001
*U/P*_osm_ T4	2.0±0.4	2.6±0.5	<0.001
Arterial pH T0	7.36±0.04	7.37±0.04	0.39
Arterial pH T4	7.34±0.03	7.35±0.03	0.18
HCO_3-_ T0, mEq/L	24±1.3	27± 3.8	0.005
HCO_3-_ T4, mEq/L	23±1.6	24±2.9	0.03
*U*_pH_ T0	6.2±0.6	5.6±0.4	0.002
*U*_pH_ T4	5.4±0.6	5.0±0.3	0.02
Urinary concentration deficit	17 (85%)	5 (29.4%)	0.001
Urinary acidification deficit	9 (45%)	1 (6.7%)	0.03

Values are expressed as mean ± SD. Significant P <0.05 *vs* control by Student *t* test. *U*_osm_, urinary osmolality after DDAVP; *U/P*osm, urinary to plasma osmolality ratio; HCO_3-_, serum bicarbonate; *U*pH, urinary pH.

**Table 4:** Comparison of renal function in hepatosplenic schistosomiasis patients and controls.

	Hepatosplenic schistosomiasis (*n* = 20)	Controls (*n* = 17)	*P*
P_Ur_, mg/dL	23±7.7	26±8.0	0.38
P_Cr_, mg/dL	0.6±0.1	0.9±0.2	0.0001
GFR, mL/min/1.73 m^2^	130±38	103±16	0.01
Microalbuminuria, mg/day	35.8±83.6	5.5±5.3	0.12
Proteinuria, mg/day	139±148	95±61	0.26
Urinary MCP-1, pg/mg-Cr	122±134	40±28	0.01
FE_Na+_, %	0.69±0.3	0.62±0.1	0.40
FE_Mg++_, %	2.96±1.8	1.81±1.0	0.14
FE_K+_, %	0.55±0.2	0.52±0.2	0.82
TTKG	3.3±1.7	3.0±1.2	0.62
Tc_H2O_, L/day	0.72±0.5	1.1±0.3	0.04

Values are expressed as mean ± SD. Significant P <0.05 *vs.* control by Student *t* test. P_Cr_, plasma creatinine; P_Ur_, plasma urea; GFR, glomerular filtration rate; MCP-1, Monocyte Chemotactic Protein-1 FE_Na+_, fractional excretion of sodium; FE_Mg++_, fractional excretion of magnesium; FE_K+_, fractional excretion of potassium; TTKG, transtubular potassium transport; TcH2O, reabsorption of free water solute.

### Hepatosplenic schistosomiasis patients have higher urinary MCP-1 in comparison with the controls

**Figure 1:**
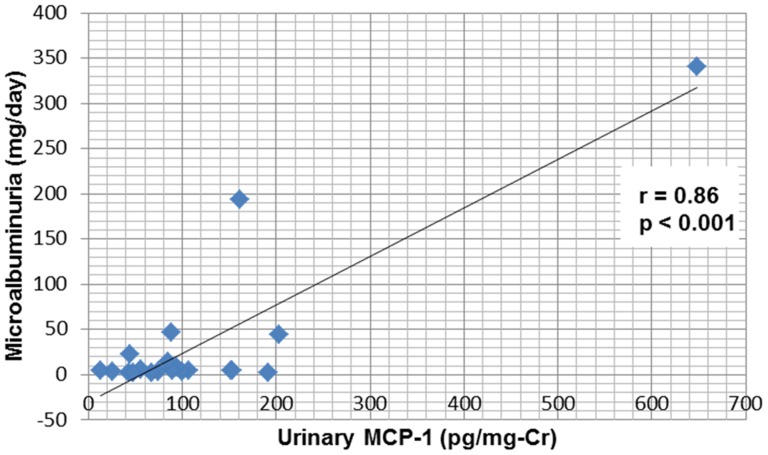
Correlation between urinary MCP-1 and albuminuria in schistosomiasis patients.

**Figure 2:**
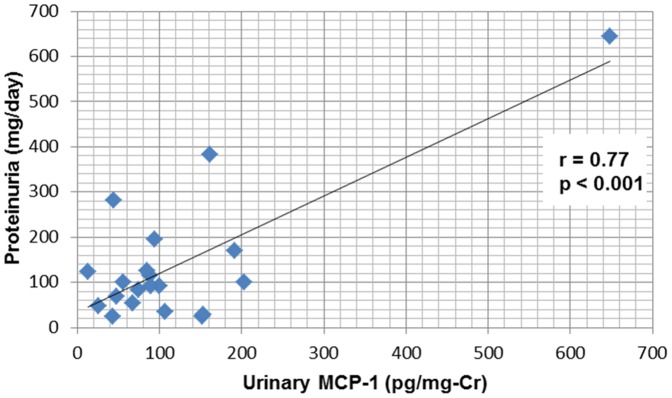
Correlation between urinary MCP-1 and proteinuria in schistosomiasis patients.

Levels of microalbuminuria and proteinuria did not differ between HSS patients and the control group. The levels of MCP-1 were higher in the HSS group than in controls (122±134 *vs* 40±28 pg/mg-Cr, *P* = 0.01) (Table 4). Abnormalities in urinary sediment were present in 20% of the 20 patients - hematuria was observed in one case (5%) and leukocyturia in 3 (15%). Microalbuminuria >30 mg/day was found in 3 cases (15%), macroalbuminuria >300 mg/day in 1 (5%) and proteinuria >150 mg/day in 5 (25%). Nephrotic-range proteinuria was not observed in any patient. MCP-1 correlated positively with microalbuminuria and proteinuria (Figs. 1 and 2).

## Discussion

The results of this cross-sectional analysis of renal function in hepatosplenic schistosomiasis clarify important aspects of tubular dysfunction in these patients and demonstrate a positive correlation between a new biomarker (MCP-1) and microalbuminuria.

The most common clinical finding related to schistosomiasis was portal hypertension. All patients had the hepatosplenic schistosomiasis form, without parasitic activity. A comparison of general laboratory tests between schistosomiasis patients and the control group did not identify significant differences, except for platelet levels, which were lower in schistosomiasis patients. This stems from the fact that although almost all patients had undergone previous splenectomy, the ones that had not undergone surgery had hypersplenism - associated thrombocytopenia.

The Child-Pugh and MELD (Model for End-Stage Liver Disease) scores were used to assess the severity and prognosis of chronic liver disease of the patients [20], [21], [24]. All patients had normal liver function tests and were classified as Child-Pugh score A. The MELD score could not be calculated because all patients had values of creatinine, INR and bilirubin within normal limits. The minimum amount required for the calculation is 1 for all three markers [24].

The hepatorenal syndrome is characterized by renal failure due to severe vasoconstriction of the renal circulation. It occurs in up to 10 percent of patients with advanced cirrhosis and ascites [25], [26]. As hepatocellular synthetic function is preserved in schistosomiasis until the onset of very advanced stages of the disease, lobular architecture is preserved and nodular regenerative hyperplasia does not occur and thus, hepatorenal syndrome is not observed. Hepatic failure in schistosomiasis is transient. Hepatic decompensation usually occurs after an episode of gastrointestinal bleeding [11], [27], [28], [29]. Although the patients had esophageal varices, we excluded patients who had had a bleeding episode in the last six months.

In our study, hypergammaglobulinemia was present in 65% of patients, which is higher than that described in literature [5] and hypocomplementemia was observed in 50%. In schistosomal nephropathy, glomerular injury may initially be asymptomatic or manifest only by hypocomplementemia. The behavior of complement is variable and may be decreased in 45 to 55% of cases [5]. Martinelli *et al*. [30] studied the serum complement in patients with histological diagnosis of schistosomal glomerulonephritis and found hypocomplementemia in 83% of patients. In the present study, there was no correlation between complement levels and renal function parameters, especially proteinuria and microalbuminuria, corroborating the idea that the hypocomplementemia may be an initial manifestation of schistosomal nephropathy.

The glomerular filtration rate was higher in HSS group compared with the control group. No patient was malnourished, which could explain this finding. Thus, there were a large number of HSS patients with glomerular hyperfiltration (40%). The main long-term consequence of glomerular hyperfiltration is the reduction of GFR due to glomerulosclerosis. No patient had reduced GFR below 60 mL/min/1.73 m^2^. The prevalence of chronic kidney disease (CKD) in patients with schistosomiasis is unclear. Martinelli *et al.* [31], in a study of renal biopsies from 21 patients with hepatosplenic schistosomiasis, found that the progressive nature of kidney disease was not influenced by therapy with anti-parasitic drugs or immunosuppressants.

For the first time, we have shown that schistosomiasis infection may present with renal tubular dysfunction demonstrated by the loss of the ability of urinary acidification and concentration. Urinary concentrating ability defect was observed in 85% of our patients. Unlike other parasitic diseases, there is no study in literature about schistosomiasis patients' inability to concentrate urine under water deprivation conditions, although it has been investigated in other infectious diseases. Oliveira *et al*. [32] studied the urinary concentrating ability in 37 patients with American Cutaneous Leishmaniasis (ACL) before and after treatment with pentavalent antimonial and found a prevalence of 77% of urinary concentrating defect, not reversed after treatment. Chugh *et al*. [33] found urinary concentrating defect and an impaired acidifying mechanism in 9 of 36 leprosy patients (25%), after an 18-h period of water deprivation. Ponce *et al*. [34] found urinary concentrating defect in 6 of 9 leprosy patients (66%). The process of urinary concentration requires the collecting duct to be intact. In this study, we also found lower levels of T_C_H_2_O (solute free water reabsorption) in HSS patients, showing a deficit in water reabsorption, which is associated with the urinary concentration defect.

In the present study, the mean values of UpH were higher in HSS patients than controls. UpH T4>5.5 was present in 45% of cases, suggesting distal tubular acidosis. There are no reports in the literature on defects in urinary acidification in schistosomiasis. This abnormality has been described in other parasitic diseases, such as cutaneous leishmaniasis, in which urinary acidification defect was found in 40% of patients before treatment and 16% after treatment, suggesting a significant improvement in acidification ability after specific treatment [32]. In visceral leishmaniasis (kala-azar), urinary acidification defect was reported in 64% of cases after treatment [35]. Drutz and Gutman [36] studied 49 leprosy patients and found that urine pH did not decrease below 5.5 in response to NH4Cl in 20% of cases. Other studies also found urinary acidification defect in leprosy patients, with variable incidence [34], [37].

Proteinuria is a common characteristic in schistosomiasis. Sobh *et al* [38] found proteinuria by dipstick in 20% of 240 ambulatory asymptomatic patients with schistosomiasis. In another study, proteinuria was detected in 24.7% of 89 patients with hepatosplenic schistosomiasis and in only 4.6% of 86 patients with the hepatointestinal form of the disease [39]. In the present study, only hepatosplenic patients were included, and proteinuria levels higher than 150 mg/day was found in 25% of cases. No patient had nephrotic-range proteinuria. Hematuria was observed in only one case (5%) and pyuria in 3 (15%).

Microalbuminuria is a known early predictor of glomerular lesion in patients with diabetes and cardiovascular diseases [40]–[42]. This change has been described in infectious diseases, but it is not yet a well-defined marker of glomerular dysfunction [43]. Elnojomi *et al* [44] detected abnormal microalbuminuria levels in 40% of patients with leishmaniasis without glomerular dysfunction. In another study of American cutaneous leishmaniasis, Oliveira *et al* [32] found abnormal microalbuminuria in 35% of patients before treatment and in only 8% after treatment, suggesting that glomerular injury can be caused by the parasitic disease itself. In leprosy, Oliveira *et al* [45] identified the presence of microalbuminuria in 8.5% of multibacillary patients. A higher prevalence of microalbuminuria was found in another study involving patients with leprosy, which identified microalbuminuria higher than 20 mg/l in 15.8% of 96 patients with leprosy [46]. In the present study, microalbuminuria >30 mg/day was found in 15%, and macroalbuminuria >300 mg/day in 5% of patients and there was no correlation between microalbuminuria and GFR. To date, only one study evaluated microalbuminuria in schistosomiasis. This recent study compared microalbuminuria levels between treated and untreated patients infected by *S. mansoni* with a healthy control group and found no difference between them. However, although there was no difference among the three groups in relation to urinary albumin excretion rate, an increase in urinary MCP-1 was observed in patients with active or treated schistosomiasis, suggesting that infection can induce a chronic renal inflammatory status that is not resolved by the specific treatment of the offending agent [19]. In the present study, MCP-1 levels were higher in schistosomiasis patients than in the control group. Moreover, we found a positive correlation between MCP-1 levels and microalbuminuria and 24-h proteinuria in patients with schistosomiasis, suggesting a role of MCP-1 in the early detection of renal damage associated with schistosomiasis.

In other kidney disorders of metabolic (diabetes mellitus), immunological (lupus nephritis) and genetic origin (autosomal dominant polycystic kidney disease) urinary MCP-1 has been correlated with urinary albumin excretion rate, glomerular filtration rate reduction and other features of kidney injury. Hanemann *et al.* [19] made the first report of an infectious disease causing an increase in urinary MCP-1, including patients with subclinical schistosomiasis. They found increased levels of urinary MCP-1 and a positive correlation between MCP-1 and microalbuminuria [19]. Our study is the second to demonstrate this association, also with schistosomiasis, but with hepatosplenic patients. Whether this finding is limited to schistosomiasis or may be initiated by any chronic infectious state remains unknown, but it is possible to speculate that any chronic infectious disease can induce renal inflammation and, consequently, urinary MCP-1 increase [18].

A limitation of our study is that we evaluated only hepatosplenic patients and it was not possible to determine whether portal hypertension could influence the results. It was not possible to compare liver function tests between the two groups, as the control group did not undertake them. However, this does not seem to affect the results, as all patients had test results within the normal range. A comparison between patients with different clinical forms of schistosomiasis could help determine whether the findings are related to the infection itself or if other factors related to portal hypertension could be influencing it.

In conclusion, the frequency of mild glomerular dysfunction, as well as tubular dysfunction, was considerably high in our cohort of HSS patients, even without clinical manifestations. It is important to evaluate renal function in patients with schistosomiasis for early detection and treatment of complications, mainly because it is a disease that predominates in younger individuals who are at higher risk of renal function loss and should benefit from measures to slow kidney disease progression. More studies are needed to evaluate the usefulness of MCP-1 as an early biomarker for schistosomal nephropathy.
